# Spatial Distribution of Cuticular Drusen and its Association with Category-Specific Progression Risk in Intermediate AMD

**DOI:** 10.21203/rs.3.rs-8504851/v1

**Published:** 2026-01-29

**Authors:** Jianfeng Huang, Muneeswar Gupta Nittala, Giulia Corradetti, Yu-Chien Chung, Alberto Quarta, Rouzbeh Abbasgholizadeh, Ceren Soylu, Shinichiro Chujo, Swetha Bindu Velaga, Srinivas R. Sadda

**Affiliations:** 1. Doheny Eye Institute, Pasadena, California, United States; 2. Department of Ophthalmology David Geffen School of Medicine, University of California Los Angeles, Los Angeles, California, United States; 3. Department of Ophthalmology, Beijing Hospital, National Center of Gerontology, Institute of Geriatric Medicine, Chinese Academy of Medical Sciences, Beijing, P.R. China; 4. Department of Ophthalmology, Fu Jen Catholic University Hospital, Fu Jen Catholic University, New Taipei City, Taiwan; 5. Department of Ophthalmology, Mie University Graduate School of Medicine, Tsu City, Mie, Japan

## Abstract

**Objectives:**

To investigate the spatial distribution pattern of cuticular drusen using en face OCT and determine its relationship with 2-year progression of age-related macular degeneration (AMD).

**Methods:**

This study included 87 eyes from 57 participants with intermediate AMD and cuticular drusen enrolled in the Amish Eye Study who completed two years of follow-up. Multimodal imaging, including volume spectral-domain OCT, was performed. Density of cuticular drusen was quantified on en face OCT across three Early Treatment Diabetic Retinopathy Study (ETDRS) grid zones using ImageJ. K-means clustering analysis was used to categorize distribution patterns. Firth’s penalized logistic regression evaluated association between cuticular drusen distribution categories and progression to late AMD at 2 years.

**Results:**

Cuticular drusen exhibited a concentric pattern within 6x6mm macular area. Mean (SD) density was highest in central zone (6.14 (3.89) count/mm2). Cluster analysis classified eyes into predominantly central (57.5%), predominantly peripheral (32.2%), and diffuse (10.3%) categories. Over 2 years, 5 eyes progressed to late AMD, 4 of which belonged to predominantly peripheral group. Firth logistic regression demonstrated that predominantly peripheral category had significantly increased risk of AMD progression compared to low-risk groups (predominantly central and diffuse), with an odds ratio of 7.2 (95% CI: 1.2–74.2, p=0.027).

**Conclusions:**

The spatial distribution of cuticular drusen exhibits a concentric, centrally-weighted pattern. A predominantly peripheral distribution of cuticular drusen is significantly associated with progression to late AMD over two years. This quantifiable distribution pattern may serve as a novel high-risk biomarker for advanced AMD.

## Introduction

Gass et al[1] first described cuticular drusen as small, round, yellow nodules clustered in the macula and in the midperiphery of the fundus. On fluorescein angiography, the lesions were shown to have characteristic pattern described as a “stars-in-the-sky” appearance[2]. Similar to hard and soft drusen, cuticular drusen are located between the basal lamina of the retinal pigment epithelium (RPE) and the inner collagenous layer of Bruch’s membrane[3].

Cuticular drusen represent a phenotype of clinical relevance in the spectrum of AMD and may have specific genetic implications such as mutations in the CFH gene[4–8]. They are distinguished by their composition, morphology, and spatial distribution, and these characteristics were suggested to confer a unique risk for the development of GA and neovascularization in a previous study[9]. However, the impact of cuticular drusen on AMD progression remains controversial. Kai et al. indicated that individuals with the cuticular drusen phenotype had neither a higher nor lower risk of developing late AMD over 3 years and were not associated with a difference in rate of visual sensitivity decline compared with those without this phenotype[10]. Similarly, a recent study of our team found that the presence of cuticular drusen may not be the risk factor for developing atrophy within a 2-year period (manuscript under review).

The reason for these apparent discrepant results regarding the impact of cuticular drusen on AMD progression may due to differential effects of different subtypes of cuticular drusen. Currently, two different classification systems have been used for categorizing cuticular drusen, one based on OCT imaging as described by Balaratnasingam et al[9] and the other one based on colour fundus photographs (CFPs) as outlined by Sakurada et al[4]. According to the study of Sakurada et al, phenotype 2 which manifested scattered cuticular drusen, extending into the periphery and phenotype 3 which was associated with large drusen (> 200 mm) were reported to have a significantly higher risk of progression to geographic atrophy (GA) and macular neovascularization (MNV) than phenotype 1, which demonstrated a cluster of numerous small lesions, concentrated centrally and thinning out circumferentially[4].

Multimodal imaging including en face OCT provide new opportunities to more precisely and quantitively study the spatial distribution of drusenoid lesions and has provide novel insights into their pathophysiology[11, 12]. To our knowledge, previous studies have not yet used en face OCT to precisely analyse the spatial pattern of cuticular drusen. Our study was designed to investigate the spatial distribution of cuticular drusen using en face OCT and to study the relationship between different spatial patterns and the 2-year risk for progression to late AMD.

## Materials and methods

### Participants

All participants with intermediate AMD (based on Beckman criteria[13] applied to colour photographs) enrolled in the Amish Eye Study, who also had a diagnosis of cuticular drusen, were included in this study. The Amish Eye Study is a National Eye Institute (NEI)-supported clinical research investigation initiated to identify biomarkers of OCT for earlier stages of AMD in a family-based cohort, as well as to identify novel phenotypic and genetic biomarkers of AMD. Detailed descriptions of the overall Amish Eye Study including demographic and multimodal imaging features can be found in previous studies[14–16]. Written informed consent was obtained from all participants prior to enrolment. The study protocol was approved by the institutional review boards of the University of California, Los Angeles; Case Western Reserve University; the University of Pennsylvania; and the University of Miami (IRB No. IRB-15-000083). All aspects of the study adhered to the tenets of the Declaration of Helsinki.

### Imaging Protocols

Inclusion criteria consisted of eyes receiving multimodal imaging including colour fundus photography, spectral-domain optical coherence tomography, consistent with established imaging protocols for AMD assessment[14, 15]: Heidelberg Spectralis HRA + OCT (Heidelberg Engineering, Heidelberg, Germany) consisted of a 512*97 macular volume cube (97 B-scans and 512 A-scans per B-scan, ART of nine frames) over a 6 mm * 6 mm field centered on the fovea; fundus autofluorescence (FAF) using Spectralis HRA, and near infrared reflectance using Spectralis HRA/OCT. Eyes were excluded if OCT demonstrated features of late AMD, if concomitant retinal pathology other than AMD was present, or if image quality was insufficient for analysis. Poor image quality was defined as significant media opacity or a signal strength of less than 20 dB on Spectralis OCT, consistent with the quality threshold used by the Doheny Image Reading Center for acceptable images.

### Grading for cuticular drusen

Traditionally, the identification of cuticular drusen has relied on clinical examination or colour fundus photography (CFP), with fluorescein angiography (FA) demonstrating the characteristic “stars-in-the-sky” pattern produced by numerous small, yellow lesions distributed throughout the posterior pole and midperiphery. Because FA is not routinely performed in patients with intermediate AMD and was not a part of the Amish Eye Study, this modality was not available in our cohort. Instead, cuticular drusen in this study were identified by examination of all B-scans in the OCT volume, and were deemed to be present when a lesion exhibited the characteristic morphologic patterns described in prior literature[9]: (1) shallow elevations of the RPE–basal lamina (BL) complex without clearly discernible internal material; (2) the classic saw-tooth configuration with a hyporeflective internal core; or (3) broad, mound-shaped RPE–BL elevations containing a homogenous medium-hyporeflective interior. It is this relatively hyporeflective interior which creates the classic “donut-like” appearance when visualized on en face OCT. Hyper-transmission tails on OCT, which have been recognized as a characteristic feature of cuticular drusen, were also included in our diagnostic criteria. Presence or absence of cuticular drusen was evaluated by 2 independent graders (JH and CC) on the basis of multimodal imaging (FAF, OCT, CFP), though the OCT features described above were the key features used for determination. Discordant diagnosis was resolved through open arbitration, with a third senior grader (S.S) making the final decision if the initial graders could not agree.

### Annotation of cuticular drusen on en face OCT

En face OCT images were obtained using the manufacturer’s multilayer segmentation software. A 20-μm-thick slab positioned from 30 to 50 μm above Bruch’s membrane was generated. Cuticular drusen could be recognized as hyperreflective ring-like (“donut”) lesions on this slab, though the hyporeflective core could be nearly pinpoint on small lesions (in which case the lesion appeared largely as a hyperreflective dot). A previous study [17] also showed that cuticular drusen manifested as donut sign on a 40μm slab from 30-70 μm above Bruch’s membrane, though due to the hypertransmission tail, this became a reverse donut on the deeper choroidal en face slab. That previous study, however, used a different OCT device, and we empirically observed that our modified slab (as described above), best highlighted cuticular drusen, particularly for quantification. En face OCT images in this study were used as a spatial guide to facilitate the localization of individual drusen. Once the boundaries of the slab were chosen as noted above, each en face image was analysed for the distribution pattern of cuticular drusen in ImageJ. Each candidate druse was verified by inspection of the corresponding cross-sectional OCT B-scan and the coordinate positions of each druse was recorded. The position of the foveal centre was also marked and recorded using this same coordinate system. The early treatment diabetic retinopathy study (ETDRS) grid was overlaid on the 6×6 mm en face OCT image. For the purpose of this analysis, only the concentric circles were used: a central circle of 1 mm diameter, an inner circle of 3 mm diameter, and an outer circle of 6 mm diameter. These circles divide the macula into 3 regions: a central foveal region (commonly termed the foveal central subfield), an inner ring (1-3mm), and an outer ring (3-6mm) [18]. The following parameters were measured in this analysis: 1) the relative location of each druse to the foveal centre which in aggregate for an eye, provides a measure of the topographical distribution of the cuticular drusen; 2) the relative distribution density of cuticular drusen within the 3 defined regions of the macular ETDRS grid. The relative distribution density was calculated by normalizing the number of manually confirmed cuticular drusen within each ETDRS region to the physical area of that region. Thus, for each eye, the total number of drusen within the central 1-mm circle, the inner 1–3-mm ring, and the outer 3–6-mm ring was divided by the corresponding area (0.79 mm^2^, 6.28 mm^2^, and 21.21 mm^2^, respectively), yielding a density measure expressed as counts per mm^2^.

### Grading of AMD

AMD staging was determined based on the Beckman classification system[13]. Intermediate AMD was defined as the presence of large drusen (⩾125 μm) or the presence of pigmentary abnormalities in at least 1 eye. Presence of exudative macular neovascularization (MNV) and/or any geographic atrophy (GA) were deemed to be evidence of late AMD. iRORA (Incomplete Retinal Pigment Epithelium and Outer Retinal Atrophy) is defined by the Classification of Atrophy Meetings (CAM) group[19], characterized as discontinuous hypertransmission signal into the choroid, attenuation or disruption of the RPE, and overlying photoreceptor degeneration on OCT. AVLs were determined on the presence of yellowish subretinal material on colour photography corresponding to hyperreflective material bounded by the external limiting membrane or ellipsoid zone anteriorly and the RPE-basal lamina-Bruch’s membrane band posteriorly on OCT[20].

### Statistical Analysis

Continuous variables are presented as mean±standard deviation. Categorical variables are presented as counts and percentages. Group differences in categorical variables were assessed using Fisher’s exact test. Associations between clusters and AMD progression were evaluated using Firth’s penalized logistic regression, a bias-reduction approach suitable for rare binary outcomes that mitigates separation issues in conventional logistic models. Statistical analysis was performed using R software (R Foundation for Statistical Computing, Vienna, Austria). A P value of 0.05 or less was considered statistically significant.

## Results

88 eyes of 58 participants were included in this analysis. All participants except for 1 (1 eye) completed the 2-year follow up. The mean age of this cohort was 70.3 ±9.6 years and 34 patients were women (59.6%).

**Figure.1:**
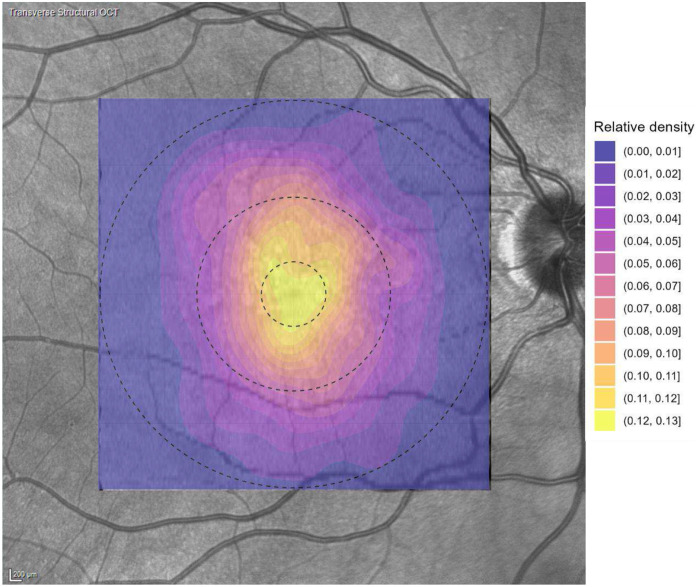
Heatmap of density of cuticular drusen.

The analysis of the density of cuticular drusen showed a concentric distribution pattern within 6*6mm macular area. As demonstrated by the quantitative assessment across the ETDRS zones, the density of cuticular drusen was inversely proportional to the distance from the foveal center, indicating a strong central concentration. The mean (standard deviation, SD) of cuticular drusen density was highest in the central zone (6.14 (3.89) count/mm^2^), significantly decreasing in the inner ring (2.15 (1.18) count/mm^2^), and lowest in the outer ring (1.18 (0.81) count/mm^2^). The detailed distribution across the three ETDRS zones is presented in Table 1 (and visually represented in Figure 1).

We applied cluster analysis with K-means cluster formula:

$$WCSS=\overset{K}{\underset{i=1}{\sum }}\underset{x\in C_{i}}{\sum }{\left\|x-\mu_{i}\right\|}^{2}$$

WCSS, the loss function or within-cluster sum of squares (WCSS), quantifies how “imperfect” the clustering is. ***K***: The number of clusters (3 in our study: predominantly central, predominantly peripheral and diffuse). *C_i_* :The set of data points belonging to the *i*-th cluster. **x** : A data point in cluster *C_i_* .In our analysis, each **x** is a single eye, represented by a vector of its 5 spatial features (mean radius, radius standard deviation, central proportion, mean nearest neighbor distance, spatial variance). ***μ***_*i*_ : The centroid of the *i*-th cluster.

**Figure.2:**
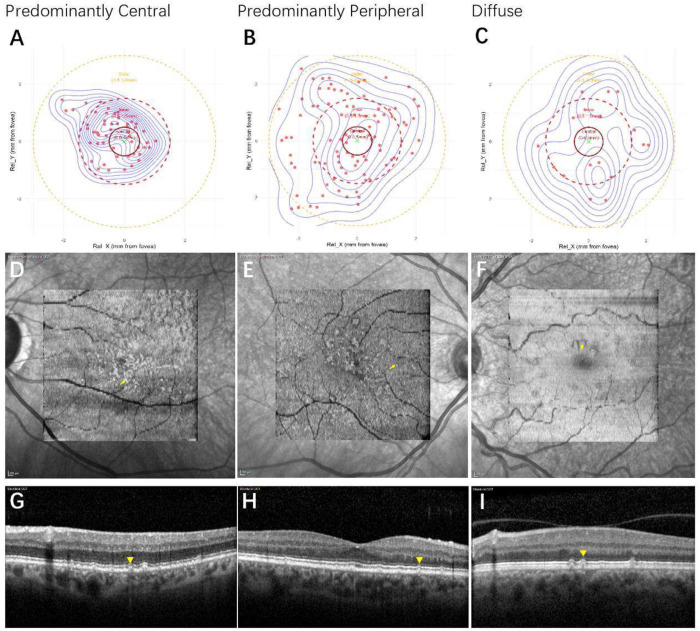
Representative figures of different groups of cuticular drusen. A-C: cluster plots of predominantly central, predominantly peripheral and diffuse group; D-F: en face images of representative eyes from predominantly central, predominantly peripheral and diffuse groups; G-I OCT B scan of eyes from predominantly central, predominantly peripheral and diffuse groups. Arrows: cuticular drusen on en face OCT; Arrow heads: cuticular drusen on OCT B scans.

According to the formula, the distribution of cuticular drusen was classified into three groups: predominantly central (50 eyes, 57.5%), predominantly peripheral (28 eyes, 32.2%), and diffuse (9 eyes, 10.3%). The predominantly central group accounted for the largest proportion. Figure 2 illustrates representative multimodal images and schematic diagrams for each group.

We evaluated proportions of baseline iRORA and AVLs, as well as the two-year progression to late AMD across the groups (Table 2). At baseline, 4 out of 87 eyes presented with AVLs, all confined to the predominantly peripheral group. During the two-year follow-up, 5 eyes developed late AMD, with 4 cases arising from the predominantly peripheral group.

Table 3 summarizes the results of pairwise Fisher’s exact tests among the three groups. At baseline, comparison between the predominantly peripheral and central groups revealed a significant difference in frequency of associated AVLs. No significant association was observed between cluster distribution patterns and iRORA at baseline. The comparison between the predominantly peripheral and central groups showed a borderline significant difference in 2-year risk of progression to late AMD.

Given that progression to late AMD primarily occurred in the predominantly peripheral group, we combined Group 1 and Group 2 as a presumed low-risk group and designated Group 3 as the high-risk group. Firth logistic regression revealed that the odds ratio for AMD progression in the high-risk group was 7.2 (95% CI: 1.2–74.2, p=0.027), which was statistically significant

## Discussion

In this study, through en face OCT analysis, we demonstrated a centrally-weighted concentric distribution pattern of cuticular drusen centred on the fovea. Within the 6*6mm macular region, the density of cuticular drusen gradually decreased from the central to the peripheral zone. Cluster analysis demonstrated that cuticular drusen distribution can be categorized into three categories: predominantly central (57.5%), predominantly peripheral (32.2%), and diffuse (10.3%). The predominance of the central category corresponds well with the overall distribution characteristics of cuticular drusen. Progression to late AMD including GA and MNV in 2 years, however, was correlated with cuticular drusen distributed in a predominantly peripheral group. To our knowledge, this study was the first to apply en face OCT to describe the distribution of cuticular drusen and its relationship of progression of AMD. The differential risk for progression based on the distribution may help explain inconsistencies with regards to progression risk in prior studies.

Previous studies[4, 9, 21] have used multimodal imaging for the diagnosis of cuticular drusen. On fundoscopy, cuticular drusen are yellowish small (25-75 μm in diameter), round, slightly raised yellow-white sub-RPE deposits[22]. On fluorescein angiography, cuticular drusen appear as a large group of small hyperfluorescent lesions scattered throughout the fundus, resulting in the “stars-in-the-sky” pattern that is characteristic of these drusen[2]. Cuticular drusen can also be visualized as inhomogeneous areas of increased reflectance on Infra-red (IR) image [23] whilst on autofluorescence, they may be recognized as focal hypoautofluorescent lesions with a hypertransmission ring[24, 25]. OCT has become a valuable tool for detection of cuticular drusen. “Typical” cuticular drusen presented as a “sawtooth” RPE elevation on OCT[2, 25], however, the saw-tooth pattern should not be considered pathognomonic, as it is not discernible in all cases[26]. Balaratnasingam et al[9] demonstrated different patterns of cuticular drusen on OCT as shallow elevations of the RPE-basal laminar band and broad, mound-shaped elevations of the RPE-basal laminar band. In our study, because participants in AMISH Eye Study had no FA acquisitions, this imaging modality was not available for grading the presence of cuticular drusen. Instead, cuticular drusen were identified in this study based on the presence of characteristic changes on OCT B-scans. The hypertransmission tail or the “barcoding” appearance into the choroid is a characteristic feature of cuticular drusen demonstrated in previous studies[10, 26, 27]. Although hypertransmission tails are not observed in all cuticular drusen, we restricted our analysis to the subset with tails to ensure high diagnostic certainty for distribution analysis.

Topographic distribution of cuticular drusen was described by Balaratnasingam et al[9] as either macular, defined as drusen distributed only within the major vascular arcades, or diffuse, defined as drusen involving the macula, but also extending beyond the vascular arcades. They found out that the distribution pattern was macular in 79 eyes (32.9%) and diffuse in 161 eyes (67.1%). In addition, their study revealed peripheral drusen anterior to the vortex veins in only 21.8% of 110 eyes with macular cuticular drusen. Shin et al.[28] had demonstrated similar distribution results in defining that the prevalence of the diffuse type in Korean patients was 61.7%. Sakurada et al [4] categorized their cuticular drusen cohort into 3 subtypes, concentrated, scattered and mixed, with 44.4%, 22.2% and 33.3% eyes of each type, respectively. Unlike the previous studies, we focused on the distribution of cuticular drusen within the macula itself, and evaluate the distribution quantitatively rather qualitatively. Though it was a laborious process, we labelled the number and position of each druse on the en face image, to allow for a more objective description of the distribution within the macula.

Whether cuticular drusen are risk factor for progression to late AMD has been a topic of controversy. Kai et al. stated that individuals with the cuticular drusen phenotype had neither a higher nor lower risk of developing late AMD over 3 years[10]. A similar result was reported in another study[29] in which cuticular drusen were not found to be a risk factor for the progression of early AMD to atrophy. On the other hand, basal laminar drusen were found to be associated with advanced AMD in a study which included a large group of eye-bank eyes[30]. Nam SW et al. demonstrated that cuticular drusen with broad, mound-shaped elevations of the RPE-basal laminar band with hyporeflective internal contents showed a significant reduction in retinal sensitivity[31]. Another study noted that eyes with both cuticular drusen and RPD may have a higher risk of developing late AMD[32]. A recent analysis by our team (manuscript under review). found that at least through 2 years, cuticular drusen did not independently increase the risk for developing late AMD. By identifying distinct subgroups of different distribution pattern, however, we were able to uncover that cuticular drusen with a predominantly peripheral pattern within the macula might be a risk factor for developing late AMD. One possible explanation is that a more peripheral distribution pattern indicates RPE dysfunction affecting a larger retinal area, and this may carry a higher risk for progression.

We also evaluated iRORA and AVLs at baseline as covariates in the analysis of AMD progression risk. A significant difference in presence of associated AVLs was observed between the predominantly peripheral and central groups. Pairwise Fisher’s exact tests at baseline, however, showed no significant association between cluster distribution patterns and iRORA. AVLs were among the first clinical associations described for cuticular drusen[33]. Reported prevalence of acquired vitelliform lesions in patients with cuticular drusen has varied across ethnic groups, ranging from 1.2% in Asian populations to 24.2% in Caucasians.[9, 28] In our study, AVLs accounted for 4.6% of the entire cohort and were exclusively concentrated in the predominantly peripheral group.

Limitations of our study include the retrospective design and a relatively small sample size. To validate our conclusions regarding the risk of late AMD, future investigations will necessitate a longer follow-up period with larger cohorts. Furthermore, the manual labelling of cuticular drusen introduces the potential for subjective bias. The future implementation of automated analysis algorithms will be essential for mitigating this potential source of error and concurrently reducing the required workload. Our methodology involved setting the specific thickness and boundaries of the en face slab based on a subjective assessment to achieve optimal visualization of cuticular drusen. Additional studies using different segmentation slabs will be important to further validate our findings. Finally, the current study was limited to analyzing the distribution of cuticular drusen within the macular area due to the use of a standard 6*6mm OCT scanning range. Subsequent research utilizing wide-field OCT is warranted to analyze distribution patterns across a broader area of the retina, and to determine who these broader patterns might influence progression risk.

## Conclusions

Our study demonstrated that the spatial distribution of cuticular drusen exhibited a concentric pattern, with a significantly higher density observed in the central macula. Using cluster analysis, we identified three distinct distribution categories—predominantly central, predominantly peripheral, and diffuse—with the predominantly central pattern being the most common. Importantly, the predominantly peripheral distribution pattern was significantly associated with progression to late AMD over two years, suggesting that this distribution pattern categorization may serve as a novel, quantifiable high-risk biomarker for advanced AMD.

## Supplementary Material

This is a list of supplementary files associated with this preprint. Click to download.

• Table2ProportionofiRORAAVLsandAMDprogressionindifferentgroups.xlsx

• Table3PairwiseFisherXXXsexacttestsamongthethreegroups.xlsx

• Table1CuticulardrusendensityindifferentETDRSzones.xlsx

Tables are available in the Supplementary Files section.

## Summary

### What was known before

- Cuticular drusen are a distinct AMD phenotype, but their impact on the risk of disease progression remains controversial.
- Previous classifications of cuticular drusen have been largely qualitative, lacking precise quantitative analysis of their spatial distribution.

### What this study adds

- This study uses en face OCT and cluster analysis to identify three distinct spatial patterns of cuticular drusen: predominantly central, predominantly peripheral, and diffuse.
- A predominantly peripheral cuticular drusen distribution was identified as a significant risk factor for 2-year progression to late AMD, suggesting that this distribution pattern categorization may serve as a novel, quantifiable high-risk biomarker for advanced AMD.
